# Volumetric modulated arc therapy versus tomotherapy for late T-stage nasopharyngeal carcinoma

**DOI:** 10.3389/fonc.2022.961781

**Published:** 2022-08-08

**Authors:** Qian Chen, Lingwei Tang, Zhe Zhu, Liangfang Shen, Shan Li

**Affiliations:** ^1^ Department of Oncology, Xiangya Hospital, Central South University, Changsha, China; ^2^ National Clinical Research Center for Geriatric Disorders, Xiangya Hospital, Central South University, Changsha, China; ^3^ College of Engineering and Management, Pingxiang University, Pingxiang, China

**Keywords:** Nasopharyngeal carcinoma, tomotherapy, VMAT, dosimetry, clinical outcomes

## Abstract

**Purpose:**

To compare the dosimetric parameters and clinical outcomes between volumetric modulated arc therapy (VMAT) and tomotherapy for treating late T-stage nasopharyngeal carcinoma (NPC).

**Methods:**

Patients with non-metastatic late T-stage NPC who received definitive radiotherapy with tomotherapy or VMAT were selected. 1:1 propensity score matching (PSM) was used to control the balance of confounding factors. The dosimetric parameters and clinical outcomes were compared.

**Results:**

A total of 171 patients were enrolled before matching, with 61 patients in the VMAT group and 110 patients in the tomotherapy group. In the post-PSM cohort, 54 sub-pairs of 108 patients were included after matching. Tomotherapy was superior to VMAT in the dosimetric parameters of planning target volumes, brainstem, spinal cord, lenses, and parotid glands but inferior in the optic nerves and optic chiasm. The tomotherapy group had a lower incidence of grade ≥ 3 acute mucositis (22.2% vs. 40.7%, p = 0.038) and a higher rate of complete response (83.3% vs. 66.7%, p = 0.046) after radiotherapy. However, there were no significant differences in locoregional failure-free survival (p = 0.375), distant metastasis-free survival (p = 0.529), or overall survival (p = 0.975) between the two groups.

**Conclusion:**

Tomotherapy is superior to VMAT in terms of most dosimetric parameters, with less acute mucositis and better short-term efficacy. There are no significant differences in the survival outcomes between the VMAT and tomotherapy groups.

## Introduction

Radiotherapy has been the primary treatment option for non-metastatic nasopharyngeal carcinoma (NPC) due to the complicated anatomical location and high tumor radiosensitivity (1). With radiotherapy techniques development, the tumor control rate and treatment side effects have improved significantly over the past three decades (2). However, radiotherapy is still very challenging for late-T stage NPC because the primary tumor is very close to several important organs at risk (OARs), such as the spinal cord, brainstem, temporal lobes, optic chiasm, and optic nerves (3). How to balance the dose requirements for the coverage of tumor target volumes and dose constraints for the protection of OARs remains a challenging issue for treating NPC patients with late-T stage.

Volumetric modulated arc therapy (VMAT) and tomotherapy are the most advanced radiotherapy modalities for treating NPC in clinical practice. VMAT is characterized by the modulated gantry rotation speed and dose rate, and tomotherapy is characterized by its rotational radiation delivery and movable couch (4). In the treatment of NPC, previous studies have shown that tomotherapy is superior or equivalent to VMAT in most dosimetric parameters, including the dose coverage of target volumes and the protections of the spinal cord, brainstem, temporal lobes, and parotids. On the other hand, VMAT has been reported to have significant advantages in shortening treatment time and protecting optic nerves and optic chiasm (4–7). Besides, the hybrid IMRT/VMAT, which combines IMRT and VMAT, has shown the potential benefit of improving the dose distribution by increasing the freedom to find the optimal combination of angular sampling and intensity modulation (8, 9). Another hybrid VMAT technique, which combines open field and VMAT, has also been reported to result in superior dose distribution and OAR protections in certain circumstance (10, 11). Noteworthy, compared with these hybrid VMAT techniques, simple VMAT has advantages in lowering MUs and shortening delivery time.However, as most of these studies were based on regenerating a radiotherapy plan with either VMAT or tomotherapy in the same patient, comparisons of radiotherapy toxicities and clinical outcomes are not available.

It is noteworthy that tomotherapy is more expensive than VMAT because of the higher costs of the equipment and longer treatment time for each patient (12). Moreover, medical insurance does not cover tomotherapy in many circumstances. As a result, the choice between VMAT and tomotherapy is a practical issue in the treatment of NPC, especially for late-T stage patients, for whom the protections of OARs are very challenging. Whether the dosimetric advantages of tomotherapy can result in reduced toxicities and better clinical outcomes is a critical concern for making choices. However, few publications have compared the clinical outcomes between VMAT and tomotherapy for NPC, especially in combination with dosimetric comparisons. Therefore, this study aimed to evaluate the differences in both dosimetric parameters and survival outcomes between VMAT and tomotherapy in treating patients with late T-stage NPC using the propensity score matching (PSM) method to control confounding factors’ balance.

## Materials and methods

### Patient enrollment

The inclusion and exclusion criteria are listed in **Table 1**. A total of 171 patients were enrolled, with 61 patients in the VMAT group and 110 patients in the tomotherapy group. The demographics of the enrolled patients are shown in **Table 2**. This study was approved by the ethics committee of our institute, and written informed consent for study inclusion was obtained from each patient.

**Table 1:** Inclusion and exclusion criteria for patient enrollment.

Inclusion criteria	Exclusion criteria
* Pathologically confirmed non-keratinizing undifferentiated or differentiated NPC * Late T-stage NPC (T3-4N0-3M0, staged or restaged according to the AJCC 8th staging system) * Patients who received induction chemotherapy followed by definitive concurrent chemoradiotherapy * The radiotherapy modality was VMAT or tomotherapy * The radiotherapy plan was available for review * Patients who received treatment at our hospital from November 2016 to December 2020	* Early T-stage or metastatic NPC (T1-2N0-3M0 or T1-4N0-3M1, staged or restaged according to the AJCC 8th staging system) * Patients who did not receive induction chemotherapy * Patients who received palliative radiotherapy * The radiotherapy modality was not VMAT or tomotherapy * The radiotherapy plan was not available for review * Patients who received treatment before November 2016

VMAT, volumetric modulated arc therapy.

**Table 2:** Demographics of the enrolled patients.

	Number of patients (%)
Age	
≤ 50 yrs	96 (56.1%)
> 50 yrs	75 (43.9%)
Sex	
Female	42 (24.6%)
Male	129 (75.4%)
EBV-DNA level	
≤ 4,000 IU/mL	156 (91.2%)
> 4,000 IU/mL	15 (8.8%)
T stage	
T1	0 (0%)
T2	0 (0%)
T3	76 (44.4%)
T4	95(55.6%)
N stage	
N0	5 (2.9%)
N1	46 (26.9%)
N2	77 (45.0%)
N3	43 (25.1%)
Clinical stage	
I	0 (0%)
II	0 (0%)
III	50 (29.2%)
IVa	121 (70.8%)
Radiotherapy modality	
VMAT	61 (35.7%)
Tomotherapy	110 (64.3%)

VMAT, volumetric modulated arc therapy.

### Treatments

All patients received induction chemotherapy (IC) for 2–3 cycles before concurrent chemoradiotherapy. The IC regimens included cisplatin plus docetaxel (DP) or cisplatin plus gemcitabine (GP). For concurrent chemotherapy, cisplatin was administered every three weeks for 2–3 cycles at a dose of 100 mg/m^2^.

For radiotherapy, simulation computed tomography (CT) and magnetic resonance imaging (MRI) were performed. The rigid fusion method was used to fuse the images of CT and MRI. The delineation of target volumes and OARs was contoured by a medical team consisting of radiation oncologists and radiologists. The principles of delineating target volumes were consistent with the international guideline for the delineation of the clinical target volumes for nasopharyngeal carcinoma (13). GTVnx and GTVnd were defined as the gross tumor volumes of the nasopharynx and lymph nodes, respectively. Clinical target volume 1 (CTV1) was delineated by combining the whole nasopharynx and a 5–10 mm expansion of the GTVnx. Clinical target volume 2 (CTV2) included the potentially involved structures and drain regions of the lymph nodes. PGTVnx, PGTVnd, PTV1, and PTV2 referred to the planning target volumes of the corresponding structures. The planning OAR volumes (PRVs) of lens, spinal cord, and brain stem were contoured by an expansion of 5, 5, and 1 mm of the corresponding structures. The prescription doses were set as follows: PGTVnx (69.96 to 70.4 Gy); PGTVnd (69.96 to 70.4 Gy); PTV1(60.06 to 60.8 Gy); and PTV2 (50.4 to 54 Gy).

### Radiation treatment planning

Varian Trilogy linear accelerator system (Varian Medical Systems, Palo Alto, CA) and Tomotherapy TomoHD system (Accuracy Inc., Sunnyvale, CA) were used to perform VMAT and tomotherapy, respectively. An Eclipse treatment planning system (RapidArc version 13.5, Varian Medical Systems, Palo Alto, CA) and a tomotherapy planning workstation (TomoHD version 2.0.7, Accuracy Inc., Sunnyvale, CA) generated the VMAT and tomotherapy plans, respectively. Dose optimizations were performed with the analytical anisotropic algorithm and the convolution/superposition algorithm in VMAT and tomotherapy planning, respectively. For VMAT, the planning parameters were set as follows: beam arrangement = 181^∘^–179^∘^ or 179^∘^–181^∘^, number of arcs = 2, collimator rotation = 0–15^∘^, gantry rotation speed = 4.8deg/s, and beam energy = 6 MV. For tomotherapy, the planning parameters were set as follows: pitch = 0.287, modulation factor = 2.0–2.6, field width = 2.5cm, and beam energy = 6 MV. During the optimization of radiation treatment planning, the priorities of the structures were divided into four levels: level 1 (brain stem and spinal cord), level 2 (PTVs), level 3 (optic nerves, optic chiasm, and temporal lobes), and level 4 (lenses and parotid glands).

### PSM

1:1 PSM was performed to balance the confouding factors that may affect the dosimetric parameters between the VMAT and tomotherapy groups. Matching covariates included PGTVnx volume, PGTVnd volume, T stage, and N stage, which have been reported to be the key factors associated with dosimetric parameters (14–16).

### Comparisons of dosimetric parameters

For evaluating the dose coverage of the PTVs, the following dosimetric parameters were adopted: conformity index (CI), homogeneity index (HI), and minimum coverage dose of 95% of the target (D95). The calculations of HI and CI were described in a previous study (7). To investigate the differences between VMAT and tomotherapy in protecting OARs, the followings parameters were compared between the two groups: the maximum dose (Dmax) and the maximum dose encompassing 1cc (D1cc) of the spinal cord, brainstem, spinal cord PRV, and brainstem PRV; the Dmax of optic nerves, optic chiasm, temporal lobes, and lenses PRV; and the relative volume receiving more than 30 Gy (V30Gy) and the mean dose (Dmean) of the parotid glands (17). Univariate and multivariate analyses were used to confirm the influence of the radiotherapy modality on dosimetric parameters. The matching covariates of PSM and the radiotherapy modality were included in the univariate analysis, and the variables demonstrating α < 0.1 were selected for the subsequent multivariate analysis.

### Comparisons of clinical outcomes

Data on acute radiotherapy side effects were retrospectively collected from the medical records, which were evaluated according to the toxicity criteria of the Radiation Therapy Oncology Group (RTOG) and the European Organization for Research and Treatment of Cancer (EORTC) (18). The Response Evaluation Criteria in Solid Tumors (RECIST) 1.1 was adopted to compare the short-term efficacy between the VMAT and tomotherapy groups, which referred to the treatment response evaluated at three months after the completion of radiotherapy. Locoregional failure-free survival (LRFS), distant metastasis-free survival (DMFS), and overall survival (OS) were calculated to estimate survival outcomes, which were defined as the length of time from the start of radiotherapy to locoregional failure, distant failure, and death. Kaplan-Meier curve analysis was used to generate LRFS, DMFS, and OS survival curves. The univariate analysis of survival outcomes included a panel of covariates that may affect patient survival in NPC, including age, sex, EBV-DNA level, pathology, T stage, GTVnx volume, N stage, GTVnd volume, induction chemotherapy regimen, concurrent cisplatin dose, adjuvant chemotherapy, and RT modality (19–22). Variates demonstrating α < 0.2 and RT modality were included in the subsequent multivariate analysis.

### Statistical analyses

The Statistical Package for the Social Sciences software program (version 25, IBM Corporation, Armonk, NY, USA) was used to perform statistical analyses. For the comparisons of baseline characteristics in the pre- and post-PSM cohorts, the chi-square test and independent t-test were used. For comparing the dosimetric parameters, an independent t-test was adopted. A linear regression model performed univariate and multivariate analyses of the dosimetric parameters. The log-rank test evaluated the significance of the differences in survival outcomes. The Cox proportional hazards model was used to perform univariate and multivariate analyses of the survival outcomes.

## Results

### Comparisons of dosimetric parameters

**Figure 1:**
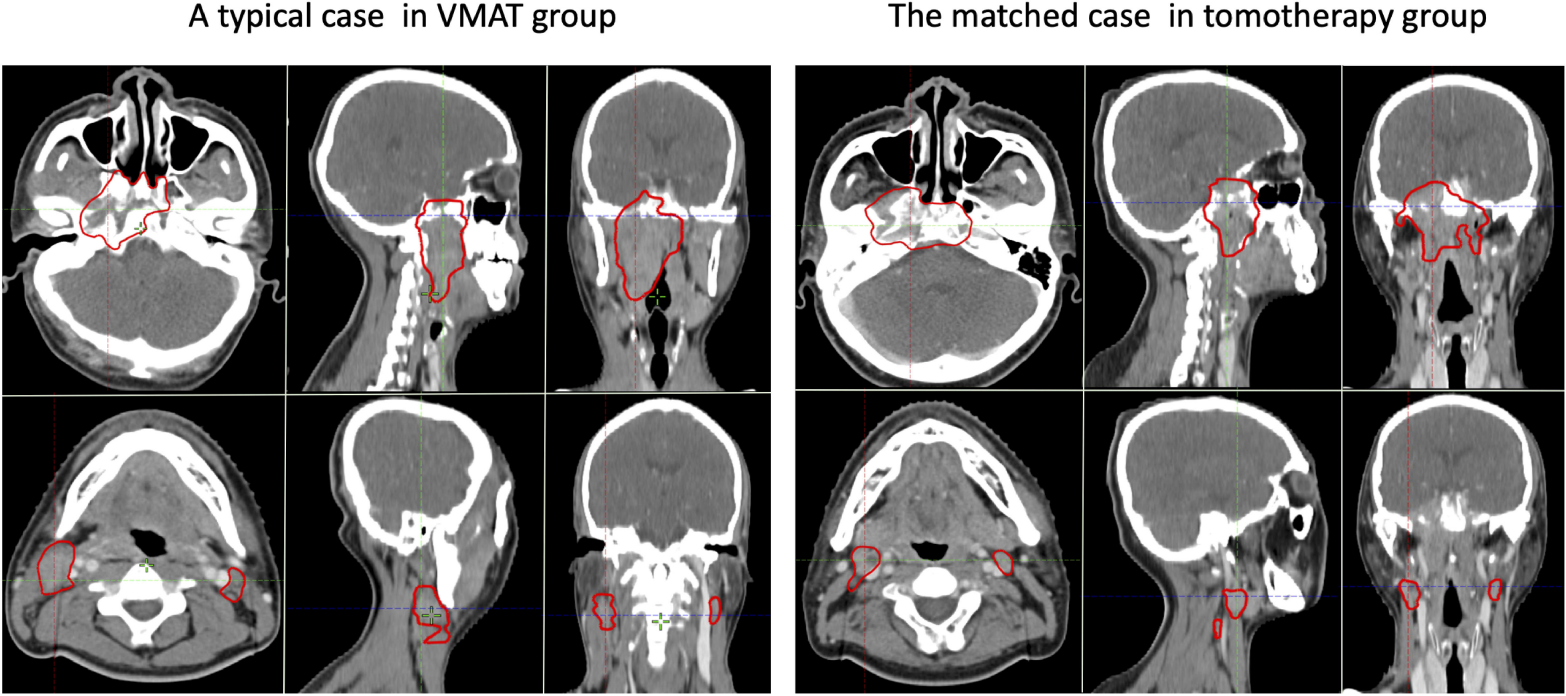
A typical pair of matched cases from the VMAT group (left) and tomotherapy group (right). The upper and lower rows show the GTVnx and GTVnd, respectively.

In the post-PSM cohort, 54 sub-pairs of 108 patients were included after matching. The baseline characteristics of the VMAT and tomotherapy groups in the pre- and post-PSM cohorts are presented in **Table 3**. A typical case in the VMAT group and its matched case in the tomotherapy group are shown in **Figure 1**.

**Table 3:** Baseline characteristics of VMAT group and tomotherapy group in the pre- and post-PSM cohorts .

	pre-PSM			post-PSM		
	VMAT (n = 61)	Tomotherapy (n = 110)	p	VMAT (n = 54)	Tomotherapy (n = 54)	p
T stage			0.006			0.560
T3	36	40		29	32	
T4	25	70		25	22	
N stage			0.802			0.961
N0	2	3		2	2	
N1	14	32		12	14	
N2	28	49		25	25	
N3	17	26		15	13	
PGTVnx volume (cm^3^)	119.98 ± 42.36	144.05 ± 52.93	0.003	126.74 ± 39.65	130.17 ± 37.58	0.646
PGTVnd volume (cm^3^)	87.90 ± 58.39	80.88 ± 61.94	0.470	88.53 ± 61.69	81.73 ± 57.37	0.554

VMAT, volumetric modulated arc therapy; PSM, propensity score matching; PGTVnx, planning target volume of the nasopharynx; PGTVnd, planning target volume of the lymph nodes.

As shown in **Table 4**, the tomotherapy group was superior in the CI of PGTVnx+nd (0.8373 vs. 0.7941, p = 0.000), D95 of PGTVnd (7104 vs.7045 cGy, p = 0.000), HI of PGTVnd (0.0677 vs. 0.0874, p = 0.000), CI of PTV1 (0.4345 vs. 0.3755, p = 0.004), D95 of PTV2 (5663 vs.5546 cGy, p = 0.000), HI of PTV2 (0.3092 vs.0.3379, p = 0.000), Dmax of spinal cord (3346 vs. 3738 cGy, p = 0.000), D1cc of spinal cord (3100 vs. 3485 cGy, p = 0.000), Dmax of spinal cord PRV (4219 vs. 4406 cGy, p = 0.026), D1cc of spinal cord PRV (3532 vs. 3873 cGy, p = 0.000), D1cc of brainstem (4559 vs. 4757 cGy, p = 0.001), Dmax of contralateral lens PRV (766 vs. 860 cGy, p = 0.008), V30Gy of ipsilateral parotid gland (57.14% vs. 65.23%, p = 0.005), Dmean of contralateral parotid gland (3607 vs. 4035 cGy, p = 0.000), and V30Gy of contralateral parotid gland (51.55% vs. 62.47%, p = 0.000). However, the VMAT group showed superior CI for PTV2 (0.7901 vs. 0.7682, p = 0.004), Dmax of the optic chiasm (5479 vs. 5922 cGy, p = 0.033), Dmax of the ipsilateral optic nerve (5499 vs. 6145 cGy, p = 0.004), and Dmax of the contralateral optic nerve (5054 vs. 5704 cGy, p = 0.001). Dose-volume histogram plots for a representative pair of matched VMAT and tomotherapy plans were shown in the **Supplementary Figure 1**.

**Table 4:** Dosimetric comparisons of the matched VMAT and tomotherapy groups.

	VMAT (Mean ± SD)	Tomotherapy (Mean ± SD)	p
PGTVnx_D95 (cGy)	6979 ± 140	7022 ± 112	0.085
PGTVnx_HI	0.1034 ± 0.0379	0.0969 ± 0.0265	0.309
PGTVnd_D95 (cGy)	7045 ± 52	7104 ± 71	0.000
PGTVnd_HI	0.0874 ± 0.0159	0.0677 ± 0.0150	0.000
PGTVnx+nd_CI	0.7941 ± 0.0598	0.8373 ± 0.0403	0.000
PTV1_D95 (cGy)	6320 ± 127	6284 ± 106	0.110
PTV1_HI	0.1968 ± 0.0297	0.1914 ± 0.0159	0.245
PTV1_CI	0.3755 ± 0.0954	0.4345 ± 0.1116	0.004
PTV2_D95 (cGy)	5546 ± 94	5663 ± 93	0.000
PTV2_HI	0.3379 ± 0.0250	0.3092 ± 0.0171	0.000
PTV2_CI	0.7901 ± 0.0404	0.7682 ± 0.0368	0.004
Spinal cord_Dmax (cGy)	3738 ± 331	3346 ± 317	0.000
Spinal cord_D1cc (cGy)	3485 ± 300	3100 ± 336	0.000
Spinal cord PRV_Dmax (cGy)	4406 ± 481	4219 ± 375	0.026
Spinal cord PRV_D1cc (cGy)	3873 ± 350	3532 ± 339	0.000
Brainstem_Dmax (cGy)	5293 ± 212	5278 ± 212	0.724
Brainstem_D1cc (cGy)	4757 ± 310	4559 ± 278	0.001
Brainstem PRV_Dmax (cGy)	5890 ± 215	5885 ± 241	0.918
Brainstem PRV_D1cc (cGy)	5185 ± 258	5109 ± 273	0.141
Optic chiasm_Dmax (cGy)	5479 ± 1183	5922 ± 938	0.033
Optic nerve I_Dmax (cGy)	5499 ± 1412	6145 ± 815	0.004
Optic nerve C_Dmax (cGy)	5054 ± 1205	5704 ± 613	0.001
Lens PRV I_Dmax (cGy)	935 ± 249	883 ± 400	0.435
Lens PRV C_Dmax (cGy)	860 ± 163	766 ± 194	0.008
Temporal lobe I_ Dmax (cGy)	7388 ± 375	7399 ± 279	0.848
Temporal lobe C_ Dmax (cGy)	6964 ± 401	6908 ± 474	0.512
Parotid gland I_Dmean (cGy)	4153 ± 669	3923 ± 760	0.098
Parotid gland I_V30Gy (%)	65.23 ± 15.66	57.14 ± 15.90	0.005
Parotid gland C_Dmean (cGy)	4035 ± 611	3607 ± 434	0.000
Parotid gland C_V30Gy (%)	62.47 ± 15.12	51.55 ± 10.11	0.000

VMAT, volumetric modulated arc therapy; PGTVnx, planning target volume of the nasopharynx; PGTVnd, planning target volume of the lymph nodes; PTV1, planning target volume of high-risk region; PTV2, planning target volume of low-risk region; D95, the minimum dose delivered to 95% of the PTVs; HI, homogeneity index; CI, conformity index; Dmax, maximum dose; D1cc, maximum dose encompassing 1cc of the structure; I, ipsilateral; C, contralateral; Dmean, mean dose; V30Gy, the relative volume of the structure receiving over 30Gy.

As shown in **Table 5**, radiotherapy modality was an independent factor associated with CI of PGTVnx+nd (p = 0.000), D95 of PGTVnd (p = 0.000), HI of PGTVnd (p = 0.000), CI of PTV1 (p = 0.000), D95 of PTV2 (p = 0.000), HI of PTV2 (p = 0.000), CI of PTV2 (p = 0.004), Dmax of spinal cord (p = 0.000), D1cc of spinal cord (p = 0.000), Dmax of spinal cord PRV (p = 0.018), D1cc of spinal cord PRV (p = 0.000), D1cc of brainstem (p = 0.000), Dmax of optic chiasm (p = 0.008), Dmax of ipsilateral optic nerve (p = 0.001), Dmax of contralateral optic nerve (p = 0.000), Dmax of contralateral lens PRV (p = 0.001), V30Gy of ipsilateral parotid gland (p = 0.005), Dmean of contralateral parotid gland (p = 0.000), and V30Gy of contralateral parotid gland (p = 0.000).

**Table 5:** Univariate and multivariate analyses of dosimetric parameters.

	T stage		N stage		PGTVnx volume		PGTVnd volume		RT modality	
	B	p	B	p	B	p	B	p	B	p
Univariate analysis										
PGTVnx_D95	-77.977	0.001	45.829	0.002	-0.779	0.015	0.307	0.142	42.500	0.085
PGTVnx_HI	0.023	0.000	-0.010	0.011	0.000	0.000	0.000	0.205	-0.006	0.309
PGTVnd_D95	-5.858	0.678	12.063	0.213	-0.201	0.271	-0.318	0.006	59.625	0.000
PGTVnd_HI	0.005	0.162	0.005	0.022	0.000	0.519	0.000	0.002	-0.020	0.000
PGTVnx+nd_CI	-0.015	0.177	0.004	0.555	0.000	0.882	0.000	0.609	0.043	0.000
PTV1_D95	-2.862	0.902	32.231	0.022	-0.012	0.968	0.323	0.094	-36.407	0.110
PTV1_HI	0.005	0.252	-0.005	0.104	0.000	0.045	0.000	0.362	-0.005	0.247
PTV1_CI	0.079	0.000	-0.078	0.000	0.001	0.000	-0.001	0.000	0.059	0.004
PTV2_D95	-5.591	0.796	11.544	0.389	0.492	0.077	0.228	0.206	116.780	0.000
PTV2_HI	0.006	0.260	-0.005	0.130	0.000	0.169	0.000	0.005	-0.029	0.000
PTV2_CI	-0.002	0.755	0.004	0.743	0.000	0.726	0.000	0.665	-0.022	0.004
Spinal cord_ Dmax	134.805	0.066	30.194	0.508	0.662	0.489	1.032	0.094	-392.500	0.000
Spinal cord_ D1cc	125.954	0.081	26.091	0.560	0.378	0.687	0.893	0.141	-384.963	0.000
Spinal cord PRV_ Dmax	190.146	0.025	40.823	0.441	3.242	0.003	0.879	0.221	-187.222	0.026
Spinal cord PRV_ D1cc	121.504	0.103	42.256	0.327	1.381	0.152	0.951	0.128	-340.981	0.000
Brainstem_ Dmax	62.193	0.130	-2.346	0.927	1.645	0.002	0.555	0.107	-14.444	0.724
Brainstem_ D1cc	83.735	0.164	0.289	0.994	1.857	0.016	0.728	0.150	-198.278	0.001
Brainstem PRV_ Dmax	102.743	0.019	3.742	0.892	1.876	0.001	0.221	0.554	-4.556	0.918
Brainstem PRV_ D1cc	122.580	0.017	0.148	0.996	2.490	0.000	0.671	0.123	-75.704	0.141
Optic chiasm_Dmax	1129.771	0.000	-369.329	0.004	9.562	0.000	-4.562	0.009	442.611	0.033
Optic nerve I_Dmax	1041.180	0.000	-450.376	0.001	8.084	0.006	-4.769	0.013	646.278	0.004
Optic nerve C_Dmax	653.890	0.001	-269.370	0.025	6.241	0.013	-3.508	0.032	650.000	0.001
Lens PRV I_Dmax	215.802	0.001	-100.740	0.014	3.048	0.000	-1.037	0.064	-52.148	0.434
Lens PRV C_Dmax	142.478	0.000	-68.125	0.002	1.754	0.000	-0.606	0.043	-93.759	0.008
Temporal lobe I_ Dmax	311.609	0.000	-91.244	0.020	3.035	0.000	-0.997	0.063	12.222	0.848
Temporal lobe C_ Dmax	98.898	0.247	-55.652	0.291	2.590	0.018	-0.375	0.601	-55.667	0.512
Parotid gland I_Dmean	-16.580	0.906	91.524	0.292	1.936	0.288	1.810	0.124	-230.093	0.098
Parotid gland I_V30Gy	1.456	0.647	0.838	0.670	0.051	0.210	0.025	0.346	-8.654	0.005
Parotid gland C_Dmean	-19.280	0.863	-20.147	0.769	-1.612	0.262	-0.641	0.492	-427.889	0.000
Parotid gland C_V30Gy	-1.039	0.703	0.153	0.928	-0.014	0.686	-0.008	0.739	-10.920	0.000
**Multivariate analysis**										
PGTVnx_D95	-33.906	0.241	34.620	0.032	-0.494	0.149	–	–	44.873	0.055
PGTVnx_HI	0.010	0.166	-0.005	0.170	0.000	0.004	–	–	–	–
PGTVnd_D95	–	–	–	–	–	–	-0.293	0.005	57.875	0.000
PGTVnd_HI	–	–	0.002	0.464	–	–	0.000	0.013	-0.019	0.000
PGTVnx+nd_CI	–	–	–	–	–	–	–	–	0.043	0.000
PTV1_D95	–	–	28.305	0.113	–	–	0.087	0.719	–	–
PTV1_HI	–	–	–	–	0.000	0.045	–	–	–	–
PTV1_CI	-0.001	0.956	-0.031	0.007	0.001	0.000	-0.001	0.000	0.048	0.000
PTV2_D95	–	–	–	–	0.411	0.083	–	–	115.072	0.000
PTV2_HI	–	–	–	–	–	–	0.000	0.000	-0.030	0.000
PTV2_CI	–	–	–	–	–	–	–	–	-0.022	0.004
Spinal cord_ Dmax	141.497	0.027	–	–	–	–	1.101	0.039	-377.148	0.000
Spinal cord_ D1cc	104.530	0.092	–	–	–	–	–	–	-379.156	0.000
Spinal cord PRV_ Dmax	76.091	0.404	–	–	2.902	0.015	–	–	-192.936	0.018
Spinal cord PRV_ D1cc	–	–	–	–	–	–	–	–	-340.981	0.000
Brainstem_ Dmax	–	–	–	–	1.645	0.002	–	–	–	–
Brainstem_ D1cc	–	–	–	–	1.976	0.007	–	–	-205.048	0.000
Brainstem PRV_ Dmax	–	–	–	–	1.64	0.007	–	–	–	–
Brainstem PRV_ D1cc	43.208	0.432	–	–	2.233	0.002	–	–	–	–
Optic chiasm_Dmax	978.266	0.000	99.248	0.502	4.307	0.101	-3.673	0.055	464.566	0.008
Optic nerve I_Dmax	874.116	0.001	-71.264	0.674	2.618	0.383	-2.444	0.264	663.963	0.001
Optic nerve C_Dmax	533.102	0.015	36.690	0.804	3.194	0.225	-2.735	0.154	652.785	0.000
Lens PRV I_Dmax	88.195	0.251	-19.396	0.714	2,587	0.006	-0.873	0.201	–	–
Lens PRV C_Dmax	55.794	0.145	-26.928	0.304	1.455	0.002	-0.418	0.215	-100.483	0.001
Temporal lobe I_ Dmax	243.160	0.001	25.905	0.588	1.796	0.035	-0.886	0.151	–	–
Temporal lobe C_ Dmax	–	–	–	–	2.590	0.018	–	–	–	–
Parotid gland I_Dmean	–	–	–	–	–	–	–	–	-230.093	0.098
Parotid gland I_V30Gy	–	–	–	–	–	–	–	–	-8.654	0.005
Parotid gland C_Dmean	–	–	–	–	–	–	–	–	-427.889	0.000
Parotid gland C_V30Gy	–	–	–	–	–	–	–	–	-10.920	0.000

RT, radiotherapy; PGTVnx, planning target volume of the nasopharynx; PGTVnd, planning target volume of the lymph nodes; PTV1, planning target volume of high-risk region; PTV2, planning target volume of low-risk region; D95, the minimum dose delivered to 95% of the PTVs; HI, homogeneity index; CI, conformity index; Dmax, maximum dose; D1cc, maximum dose encompassing 1cc of the structure; I, ipsilateral; C, contralateral; Dmean, mean dose; V30Gy, the relative volume of the structure receiving over 30Gy.

### Comparisons of clinical outcomes

**Figure 2:**
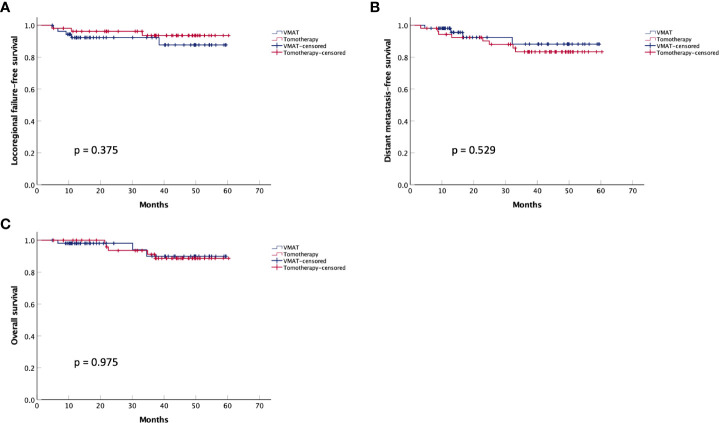
Kaplan-Meier survival curves for the matched VMAT and tomotherapy groups. **(A)** Locoregional failure-free survival, **(B)** Distant metastasis-free survival, and **(C)** Overall survival.

Regarding acute toxicities (grade ≥3), the tomotherapy group had a lower incidence of mucositis (22.2% vs. 40.7%, p = 0.038), as shown in **Table 6**. For short-term efficacy, the tomotherapy group achieved a higher complete response rate (83.3% vs. 66.7%, p = 0.046) three months after the completion of radiotherapy, as shown in **Table 7**. The median follow-up time was 38 months. As shown in **Figure 2**, there were no significant differences in LRFS (p = 0.375), DMFS (p = 0.529), or OS (p = 0.975) between the two groups. As shown in **Supplementary Tables 1–3**, multivariate analysis showed that the radiotherapy modality was not an independent factor associated with LRFS (p = 0.327), DMFS (p = 0.347), and OS (p = 0.919).

**Table 6:** Comparisons of acute toxicities (grade ≥ 3) between the matched VMAT and tomotherapy groups.

	VMAT	Tomotherapy	p
Mucositis	40.7% (22/54)	22.2% (12/54)	0.038
Dermatitis	5.6% (3/54)	3.7% (2/54)	0.647
Hematologic disorders	25.9% (14/54)	20.4% (11/54)	0.494

VMAT, volumetric modulated arc therapy.

**Table 7:** Comparisons of short-term efficacy between the matched VMAT and tomotherapy groups.

	IMRT	Tomotherapy	p
CR	66.7% (36/54)	83.3% (45/54)	
PR	33.3% (18/54)	16.7% (9/54)	
SD	0% (0/54)	0% (0/54)	
PD	0% (0/54)	0% (0/54)	0.046

VMAT, volumetric modulated arc therapy; CR, complete response; PR, partial response; SD, stable disease; PD, progressive disease.

## Discussion

Despite advances in radiotherapy techniques, treating late-T stage NPC is still very challenging because of the complicated locations of the primary tumor and nearby OARs. Although tomotherapy has been reported to have more dosimetric advantages over VMAT in treating NPC, whether it can result in superior clinical outcomes remains unclear. To the best of our knowledge, this study is the first to compare the dosimetric parameters and clinical outcomes of VMAT and tomotherapy for the treatment late-T stage NPC.

Our results showed that tomotherapy was superior to VMAT in most dosimetric parameters, including dose coverage, dose homogeneity, dose conformity of PTVs and the protection of the spinal cord, brainstem, lenses, and parotid glands in concordance with the results of previous studies (4–7). However, it should be pointed out that the tomotherapy group showed a significant shortage in protecting the optic nerves and optic chiasm, which has also been reported in several previous studies (4, 5, 7). A possible explanation for this phenomenon is that the relatively thick field width and fixed jaw of tomotherapy can lead to a craniocaudal dose spread above the treatment target (4, 7, 23). Therefore, tomotherapy may not be the best choice in situations where protection of the optic function has the highest priority unless dynamic jaws that allow for smaller jaws at the cranial and caudal parts are available (23, 24).

It is worth mentioning that most of the previous dosimetric studies make dosimetric comparisons by regenerating a VMAT/tomotherapy plan on the same patient with an existing tomotherapy/VMAT plan, which makes comparisons of clinical outcomes infeasible (4–7). The current study compared the dosimetric parameters between patients who received tomotherapy and those who received VMAT directly, using PSM to control the balance of confounding factors that may affect dosimetry. Therefore, our study’s results further confirm the dosimetric comparisons between tomotherapy and VMAT from a new perspective and provide information on clinical outcomes.

In concordance with the dosimetry advantages, the tomotherapy group demonstrated a lower incidence of acute mucositis and a higher CR rate after radiotherapy. However, there were no significant differences in LRFS (p = 0.375), DMFS (p = 0.529), and OS (p = 0.975) between the two groups, as indicated by both Kaplan-Meier curves and multivariate analyses. One possible explanation for the insignificant advantages in survival outcomes is that patients in the VMAT group were more likely to receive adjuvant chemotherapy because of the higher residual disease rates after radiotherapy, which may counteract the potential advantages of radiotherapy modality (25–27). Another possible explanation is that the inferiority in dose coverage of the PTVs in the VMAT group may not affect survival outcomes, as several studies have indicated that dose and target volume reduction did not affect the survival outcomes for NPC patients who received induction chemotherapy (28–30).

It is noteworthy that the hybrid IMRT/VMAT technique which combines IMRT and VMAT, has been reported to offer improvement in the dose coverage of target volumes and achieve better or equal protections of OARs than VMAT in NPC, non-small cell lung cancer, prostate cancer, and olfactory neuroblastoma (8, 9, 31–33). As the current study only investigated the VMAT technique, comparisons between hybrid IMRT/VMAT and tomotherapy should be explored in the future study.

This study results were potentially affected by several intrinsic limitations. First, as only 108 patients were included in the pos-PSM cohort, the sample size was relatively small. Second, late toxicities were not compared between the two groups because of incomplete records of relevant information during follow-up. Third, all patients were enrolled from our institute, which may have caused bias in the patient population. Fourth, seven patients in the VMAT group were removed because of unsuccessful matching during the PSM process, which may have led to a selection bias.

## Conclusion

Tomotherapy is superior to VMAT in terms of most dosimetric parameters, with less acute mucositis and better short-term efficacy. There are no significant differences in the survival outcomes between the VMAT and tomotherapy groups.

## Data availability statement

The original contributions presented in the study are included in the article/**Supplementary Material**. Further inquiries can be directed to the corresponding author.

## Ethics statement

The studies involving human participants were reviewed and approved by the ethics committee of Xiangya Hospital, Central South University. The patients/participants provided their written informed consent to participate in this study.

## Author contributions

SL and LS contributed to the study conception and design. Material preparation and data collection were performed by QC and LT. Data analysis were performed by QC, LT and ZZ. The first draft of the manuscript was written by QC and all authors commented on previous versions of the manuscript. All authors read and approved the final manuscript.

## Conflict of interest

The authors declare that the research was conducted in the absence of any commercial or financial relationships that could be construed as a potential conflict of interest.

## Publisher’s note

All claims expressed in this article are solely those of the authors and do not necessarily represent those of their affiliated organizations, or those of the publisher, the editors and the reviewers. Any product that may be evaluated in this article, or claim that may be made by its manufacturer, is not guaranteed or endorsed by the publisher.

## Supplementary material

Supplementary Figure 1 Dose-volume histogram plots for a representative pair of matched VMAT plan **(A)** and Tomotherapy plan **(B)**. Target volumes and OARs are represented by lines: PGTVnx, PGTVnd, PTV2, and PTV2 (blue); brainstem and brainstem PRV (orange); spinal cord and spinal cord PRV (magenta); optic nerve and optic chiasm (green); temporal lobes (brown); lenses PRV (dark green); parotids (cyan).
