# Supplementary material for: Volumetric modulated arc therapy versus tomotherapy for late T-stage nasopharyngeal carcinoma
Source: Front Oncol. 2022 Aug 8;12:961781. doi: 10.3389/fonc.2022.961781 (PMC9393424; doi:10.3389/fonc.2022.961781)
Supplement: Supplementary file 1 [file DataSheet_1.docx]

Supplementary Table 1. Univariate and multivariate analyses of LFRS

|  | Univariable analysis | |  | Multivariable analysis | |
| --- | --- | --- | --- | --- | --- |
|  | HR (95% CI) | p |  | HR (95% CI) | p |
| Age | 1.029 (0.955,1.109) | 0.456 |  | - | - |
| Sex |  | 0.354 |  |  | - |
| Male | 1.000 Reference |  |  | - |  |
| Female | 1.967 (0.470,8.234) |  |  | - |  |
| EBV-DNA level |  | 0.145 |  |  | 0.317 |
| ≤ 4,000 IU/mL | 1.000 Reference |  |  | 1.000 Reference |  |
| > 4,000 IU/mL | 3.302 (0.663,16.435) |  |  | 3.083 (0.339,28.026) |  |
| Pathology |  | 0.839 |  |  | - |
| Nonkeratinizing differentiated | 1.000 Reference |  |  | - |  |
| Nonkeratinizing undifferentiated | 0.843 (0.163,4.353) |  |  | - |  |
| T stage |  | 0.064 |  |  | 0.178 |
| T3 | 1.000 Reference |  |  | 1.000 Reference |  |
| T4 | 4.554 (0.916,22.634) |  |  | 3.424 (0.571,20.535) |  |
| Pre-treatment GTVnx volume | 1.016 (1.000,1.033) | 0.055 |  | 1.008 (0.986,1.031) | 0.464 |
| N stage |  | 0.539 |  |  | - |
| N0-1 | 1.000 Reference |  |  | - |  |
| N2-3 | 0.639 (0.153,2.676) |  |  | - |  |
| Pre-treatment GTVnd volume | 1.010 (0.990,1.030) | 0.328 |  | - | - |
| IC regimen |  | 0.298 |  |  | - |
| DP | 1.000 Reference |  |  | - |  |
| GP | 2.339 (0.472,11.602) |  |  | - |  |
| Concurrent cisplatin dose |  | 0.817 |  |  | - |
| < 200mg/m^2^ | 1.000 Reference |  |  | - |  |
| ≥ 200mg/m^2^ | 1.281 (0.157,10.430) |  |  | - |  |
| Adjuvant chemotherapy |  | 0.061 |  |  | 0.707 |
| No | 1.000 Reference |  |  | 1.000 Reference |  |
| Yes | 4.648 (0.934,23.143) |  |  | 1.569 (0.150,16.436) |  |
| RT modality |  | 0.383 |  |  | 0.327 |
| VMAT | 1.000 Reference |  |  | 1.000 Reference |  |
| Tomotherapy | 0.528 (0.126,2.220) |  |  | 0.478 (0.110,2.088) |  |

Abbreviations: LRFS = locoregional failure-free survival, GTVnx = gross tumor volume of nasopharynx, GTVnd = gross tumor volume of lymph nodes, IC = induction chemotherapy, DP = docetaxel plus cisplatin, GP = gemcitabine plus cisplatin, RT = radiotherapy, VMAT = volumetric modulated arc therapy

Supplementary Table 2. Univariate and multivariate analyses of DMFS

|  | Univariable analysis | |  | Multivariable analysis | |
| --- | --- | --- | --- | --- | --- |
|  | HR (95% CI) | p |  | HR (95% CI) | p |
| Age | 0.994 (0.941,1.049) | 0.817 |  | - | - |
| Sex |  | 0.238 |  |  | - |
| Male | 1.000 Reference |  |  | - |  |
| Female | 0.292 (0.038,2.258) |  |  | - |  |
| EBV-DNA level |  | 0.598 |  |  | - |
| ≤ 4,000 IU/mL | 1.000 Reference |  |  | - |  |
| > 4,000 IU/mL | 1.505 (0.329,6.881) |  |  | - |  |
| Pathology |  | 0.581 |  |  | - |
| Nonkeratinizing differentiated | 1.000 Reference |  |  | - |  |
| Nonkeratinizing undifferentiated | 1.534 (0.336,7.010) |  |  | - |  |
| T stage |  | 0.873 |  |  |  |
| T3 | 1.000 Reference |  |  |  | - |
| T4 | 1.098 (0.347,3.472) |  |  | - |  |
| Pre-treatment GTVnx volume | 1.006 (0.986,1.025) | 0.574 |  | - |  |
| N stage |  | 0.345 |  |  | - |
| N0-1 | 1.000 Reference |  |  | - |  |
| N2-3 | 2.080 (0.455,9.501) |  |  | - |  |
| Pre-treatment GTVnd volume | 1.013 (0.997,1.029) | 0.106 |  | 1.015 (0.998,1.033) | 0.075 |
| IC regimen |  | 0.337 |  |  | - |
| DP | 1.000 Reference |  |  | - |  |
| GP | 0.030 (0.000,38.335) |  |  | - |  |
| Concurrent cisplatin dose |  | 0.341 |  |  | - |
| < 200mg/m^2^ | 1.000 Reference |  |  | - |  |
| ≥ 200mg/m^2^ | 0.530 (0.143,1.960) |  |  | - |  |
| Adjuvant chemotherapy |  | 0.948 |  |  | - |
| No | 1.000 Reference |  |  | - |  |
| Yes | 1.071 (0.138,8.301) |  |  | - |  |
| RT modality |  | 0.531 |  |  | 0.347 |
| VMAT | 1.000 Reference |  |  | 1.000 Reference |  |
| Tomotherapy | 1.472 (0.439,4.934) |  |  | 1.832 (0.512,6.462) |  |

Abbreviations: DMFS = distant metastasis-free survival, GTVnx = gross tumor volume of nasopharynx, GTVnd = gross tumor volume of lymph nodes, IC = induction chemotherapy, DP = docetaxel plus cisplatin, GP = gemcitabine plus cisplatin, RT = radiotherapy, VMAT = volumetric modulated arc therapy

Supplementary Table 3. Univariate and multivariate analyses of OS

|  | Univariable analysis | |  | Multivariable analysis | |
| --- | --- | --- | --- | --- | --- |
|  | HR (95% CI) | p |  | HR (95% CI) | p |
| Age | 1.006 (0.939,1.079) | 0.858 |  | - | - |
| Sex |  | 0.498 |  |  | - |
| Male | 1.000 Reference |  |  | - |  |
| Female | 0.485 (0.060,3.944) |  |  | - |  |
| EBV-DNA level |  | 0.505 |  |  | - |
| ≤ 4,000 IU/mL | 1.000 Reference |  |  | - |  |
| > 4,000 IU/mL | 0.041 (0.000,493.412) |  |  | - |  |
| Pathology |  | 0.496 |  |  | - |
| Nonkeratinizing differentiated | 1.000 Reference |  |  | - |  |
| Nonkeratinizing undifferentiated | 2.070 (0.254,16.841) |  |  | - | - |
| T stage |  | 0.168 |  |  | 0.304 |
| T3 | 1.000 Reference |  |  | 1.000 Reference |  |
| T4 | 2.741 (0.654,11.490) |  |  | 2.217 (0.486,10.121) |  |
| Pre-treatment GTVnx volume | 1.003 (0.978,1.030) | 0.795 |  | - | - |
| N stage |  | 0.621 |  |  | - |
| N0-1 | 1.000 Reference |  |  | - |  |
| N2-3 | 0.697 (0.166,2.918) |  |  | - |  |
| Pre-treatment GTVnd volume | 1.006 (0.983,1.030) | 0.624 |  | - | - |
| IC regimen |  | 0.636 |  |  | - |
| DP | 1.000 Reference |  |  | - |  |
| GP | 0.033 (0.000,47485.253) |  |  | - |  |
| Concurrent cisplatin dose |  | 0.347 |  |  | - |
| < 200mg/m^2^ | 1.000 Reference |  |  | - |  |
| ≥ 200mg/m^2^ | 0.464 (0.093,2.301) |  |  | - |  |
| Adjuvant chemotherapy |  | 0.098 |  |  | 0.236 |
| No | 1.000 Reference |  |  | 1.000 Reference |  |
| Yes | 3.859 (0.778,19.135) |  |  | 2.788 (0.512,15.178) |  |
| RT modality |  | 0.975 |  |  | 0.919 |
| VMAT | 1.000 Reference |  |  | 1.000 Reference |  |
| Tomotherapy | 1.023 (0.243,4.310) |  |  | 0.928 (0.218,3.954) |  |

Abbreviations: OS = overall survival, GTVnx = gross tumor volume of nasopharynx, GTVnd = gross tumor volume of lymph nodes, IC = induction chemotherapy, DP = docetaxel plus cisplatin, GP = gemcitabine plus cisplatin, RT = radiotherapy, VMAT = volumetric modulated arc therapy
