# Supplementary figures and images for: Volumetric modulated arc therapy versus tomotherapy for late T-stage nasopharyngeal carcinoma
Source: Front Oncol. 2022 Aug 8;12:961781. doi: 10.3389/fonc.2022.961781 (PMC9393424; doi:10.3389/fonc.2022.961781)

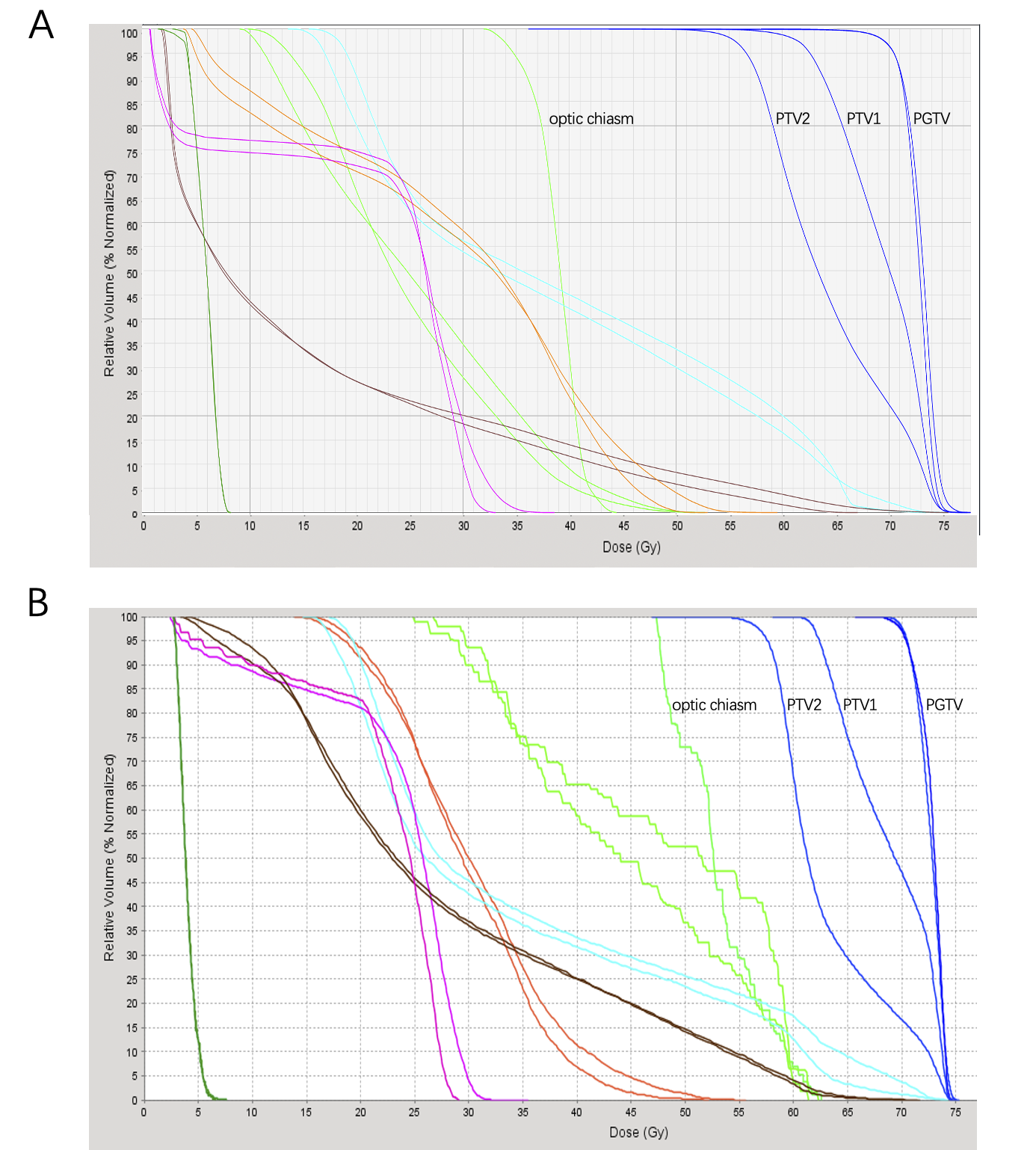

Supplement: Supplementary Figure 1 — Dose-volume histogram plots for a representative pair of matched VMAT plan (A) and Tomotherapy plan (B). Target volumes and OARs are represented by lines: PGTVnx, PGTVnd, PTV2, and PTV2 (blue); brainstem and brainstem PRV (orange); spinal cord and spinal cord PRV (magenta); optic nerve and optic chiasm (green); temporal lobes (brown); lenses PRV (dark green); parotids (cyan). [file Image_1.png]
